# Enabling selective absorption in perovskite solar cells for refractometric sensing of gases

**DOI:** 10.1038/s41598-020-63570-y

**Published:** 2020-05-08

**Authors:** Mahmoud H. Elshorbagy, Alexander Cuadrado, Beatriz Romero, Javier Alda

**Affiliations:** 1 Applied Optics Complutense Group, Faculty of Optics and Optometry, University Complutense of Madrid, 28037 Madrid, Spain; 2 Physics Department, Faculty of Science, Minia University, El Minya, 61519 Egypt; 3 Escuela de Ciencias Experimentales y Tecnología, University Rey Juan Carlos, Móstoles, 28933 Madrid Spain

**Keywords:** Optical techniques, Applied optics, Optical sensors, Solar energy and photovoltaic technology, Nanophotonics and plasmonics

## Abstract

Perovskite solar cells are currently considered a promising technology for solar energy harvesting. Their capability to deliver an electrical signal when illuminated can sense changes in environmental parameters. We have numerically analyzed the variation of the current delivered by a perovskite cell as a function of the index of refraction of air, that is in contact with the front surface of the cell. This calculation identifies which geometrical and material structures enhance this behavior. After replacing the top transparent electrode of a solar cell by an optimized subwavelength metallic grating, we find a large variation in the responsivity of the cell with respect to the change in the index of refraction of the surrounding medium. Such a refractometric sensor can be interrogated electronically, avoiding the cumbersome set-ups of spectral or angular interrogation methods. We present an adaptation of the performance parameters of refractometric sensors (sensitivity and figure of merit) to the case of opto-electronic interrogation methods. The values of sensitivity and Figure of Merit are promising for the development of refractometric perovskite-based sensors.

## Introduction

What if the front surface of a solar cell were functionalized to work as an optical sensor? This question was asked and answered a long time ago in previous published results. The answer includes materials and sensitized coatings to selectively detect some specimens and substances using solar cells. The published results report applications for optical sensing^1^, gas sensing^2,3^, refractive index sensing^4–7^, chemical sensing^8^, and multi-functional sensors^9^. The advantage is clear: the signal is directly generated by the solar cell and, ideally, there is no need for sophisticated and voluminous read-out elements (goniometers and spectrometer for angular and spectral interrogation techniques)^10–12^.

The measurement of the variation in the index of refraction is one of the most successful strategies for optical sensing of a variety of compounds^13–17^. Basically, the optical sensor becomes a refractometric sensor. This approach can be applied to self-powered sensors based on solar cells^3^. Although this side effect application of solar cells may seem exotic, we will show how nanophotonic structures boost the capabilities of perovskite-based solar cells for their use as refractometric sensors. This transformation from a solar cell to a sensor also implies a discussion to properly describe the performance of the system as a dedicated refractometric sensors, or as a solar cell.

In this contribution, we numerically analyze and propose a modified perovskite solar cell to work as a refractometric sensor for gases. Silicon solar cells could be considered as a first choice because of its stability and durability. However, in the last decade, perovskite cells have demonstrated a fast performance enhancement in terms of efficiency and durability^18–20^. Although there are some problems still to be solved (reliability, degradation, etc.)^21^, these devices are one of the most promising players in photovoltaic technologies, and perovskite solar cells are currently considered as one of the possible silicon substitutes. An interesting use for the device presented in this contribution is in environmental research applications, as well as in industrial inert atmospheres or laboratory clean rooms^22,23^. As a simple sensor fore gases, it may help to detect volatile compounds with potential environmental hazard, and to monitor air quality parameters and gas composition^24–33^. The measurement of the index of refraction of air, as an ubiquitous gas, is of interest since long ago^34–36^. Several proposals have been presented to monitor air quality and composition^23,29–31^. It is also known, that the index of refraction of air, or any other gas or gas mixture, depends on physical parameters (temperature and pressure) and chemical composition (humidity, presence of natural or artificial specimens). Then, a refractometric sensor can check if some preset conditions are fulfilled, or a known specimen varies its concentration. To do that, several optical technologies have been applied to sense refractometric changes in gases. For example, we can find interferometric systems^14,37–40^ plasmonic devices^31,41–43^, or sensor based on specialized fibers and photonic crystals^23,26,32,44,45^. Our system, based on the selective resonances generated by subwavelength metallic gratings on a modified perovskite cell can provide an alternative. The capabilities of optical gas sensors rely on the accuracy and resolution of the involved optical instruments. A high-performance sensor requires high quality optical subsystems, making the device more complex and expensive. The sensor proposed in this contribution delivers an electric signal to voltage, or current, meters, that are, by far, more available at lower cost.

**Figure 1:**
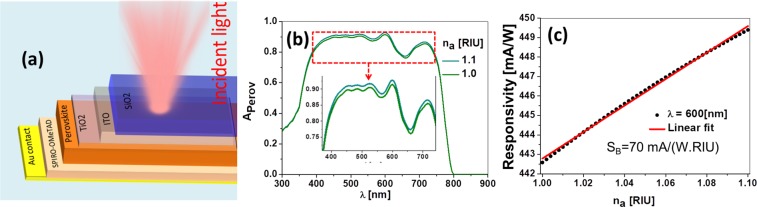
(**a**) Layer structure of a perovskite solar cell. The cell is illuminated through the SiO_2_ (glass) substrate. From top to bottom: air, glass (SiO_2_, ${t}_{{\rm{S}}i{O}_{2}}\mathrm{=3}$ mm), top electrode (ITO, ${t}_{{\rm{I}}TO}\mathrm{=70}$ nm), buffer layer (TiO_2_, ${t}_{{\rm{T}}i{O}_{2}}\mathrm{=30}$ nm), active layer (perovskite, ${t}_{{\rm{p}}{\rm{e}}{\rm{r}}{\rm{o}}{\rm{v}}{\rm{s}}}\mathrm{=300}$ nm), buffer layer (${t}_{{\rm{s}}{\rm{p}}{\rm{i}}{\rm{r}}{\rm{o}}}\mathrm{=160}$ nm), and bottom electrode (${t}_{{\rm{A}}u}\mathrm{=200}$ nm). (**b**) Spectral absorption of active layer for two indices of refraction of the outer medium, n_*a*_ = 1.0 and *n*_*a*_ =1.1. (**c**) Responsivity, *R* as a function of *n*. The sensitivity of this device is given by the slope of the linear fit: *S*_*B*_ = 70 mA/(W.RIU).

Figure 1a shows a typical layer structure used for perovskite solar cells illuminated from the substrate^46,47^. In the same figure we have also evaluated the spectral absorption for two values of the index of refraction of the media in contact with the front surface (see Fig. 1b). Our first approach is to consider air as the analyte (*n*_*a*_ = 1), and slightly change its index of refraction while we monitor the response of the system. The details of this calculation are given in the following section 2. In energy harvesting applications, the performance of the cell is parameterized with its efficiency, which is related to the short-circuit current, or the short-circuit density current, delivered by the cell, *I*_*sc*_ or *J*_*sc*_, respectively. This current is proportional to the optical absorbed power at the active layer of the cell, that also depends on the spectral composition of the radiation. In our analysis, we can numerically evaluate the absorbed power within the perovskite layer, ${A}_{{\rm{p}}{\rm{e}}{\rm{r}}{\rm{o}}{\rm{v}}}$, and therefore obtain the opto-electronic response of the cell^48^. This signal is also dependent on the index of refraction of the medium in front of the cell that becomes the analyte. Therefore, we evaluate the sensitivity of the cell as a sensor, which is defined as the change of a physical property of the device with respect to an environmental change. In this case, we use the responsivity of the cell, *R* (in mA/W), as the parameter that changes with the refractive index of the analyte, *n*_*a*_.^49,50^. In Fig. 1c we represent the change in responsivity, R, as a function of the index of refraction of the analyte when the system is illuminated at *λ* = 600 nm and 50 mW/cm^2^ in irradiance. When *I*_sc_ is used to define the sensitivity, ${S}_{B}=\partial {I}_{{\rm{s}}c}/\partial {n}_{a}$, we find a value of ${S}_{B}\mathrm{=190}$ mA/RIU (where RIU means refractive index units). However, when using responsivity, we obtain ${S}_{B}=\partial R/\partial {n}_{a}\mathrm{=70}$ mA/(W.RIU). We use responsivity because of its robustness and flexibility to define the sensitivity independently from the power of the source illuminating the cell. As it is being demonstrated, this idea shows potential that it is worth exploring. Therefore, the next step is to change the design of the cell and improve its optical sensor’s performance, while maintaining the structure of a working perovskite solar cell. This approach would allow an easy implementation, so customized solar cells could work as refractometric sensors. A similar approach has been successfully proved when adapting a CMOS device for sensing^51^, and when perovskite photodetectors are used in visible light communication systems^52^. The solar cells becomes closer to a photodetector when the delivered electric signal varies for changes in *n*_*a*_, acting like a sensor. Then, the performance parameters commonly used in sensing (sensitivity, figure of merit, FOM) may not be directly applicable here, so the capabilities of the system should be related with detection figures of merit as responsivity, noise equivalent power, detectivity, etc^52,53^.

## Materials and methods

**Figure 2:**
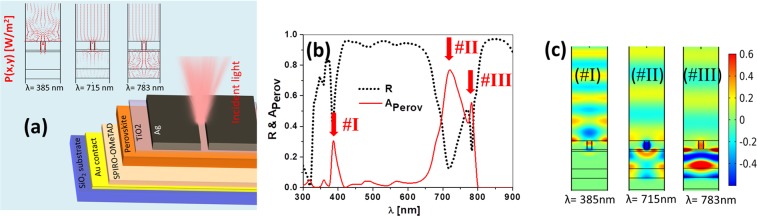
(**a**) Layer structure of a perovskite solar cell where the top electrode has been replaced by a nanostructured metallic layer having a thickness of ${t}_{{\rm{A}}g}\mathrm{=150}$ nm. A periodic grating with subwavelength period, *p* = 375 nm, and apertures, GW = 50 nm, allows light funneling towards the active layer of the cell. The inset at the left show three maps of the Poynting vector where we see how the power is directed towards different portions of the solar cell for ${\lambda }_{{\rm{I}}}$, ${\lambda }_{{\rm{I}}{\rm{I}}}$, and ${\lambda }_{{\rm{I}}{\rm{I}}{\rm{I}}}$. (**b**) Spectral reflectance of the structure (black dotted line), and spectral absorption of the active layer (red solid line). Both the spectral absorption and reflectance show three peaks (I, II, and III) at ${\lambda }_{{\rm{I}}}\mathrm{=385}$ nm, ${\lambda }_{{\rm{I}}{\rm{I}}}\mathrm{=715}$ nm, and ${\lambda }_{{\rm{I}}{\rm{I}}{\rm{I}}}\mathrm{=783}$ nm. (**c**) Electric field distribution for the same wavelengths considered in (**a**,**b**). The three resonances show their maximum amplitude at different layers within the solar cell.

**Figure 3:**
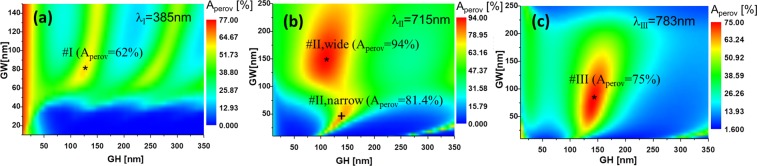
Maps of the absorption at the perovskite active layer, ${A}_{{\rm{perov}}}$, in terms of the height (GH) and width (GW) of the slits, maintaining the thickness of the metal layer, and the grating period. Each map corresponds to one of the three spectral resonances centered at ${\lambda }_{{\rm{I}}},{\lambda }_{{\rm{II}}}$, and ${\lambda }_{{\rm{III}}}$. There are two maxima for ${\lambda }_{{\rm{II}}}$, labeled as # II-wide and # II-narrow according to the width of the slit. The value of ${A}_{{\rm{perov}}}$ is given for every optimum design. We have represented these plots using colormaps with different ranges to emphasize the variations for each case. Therefore, the comparison between maps should consider the applicable ranges. The optimum designs are marked and labeled as in Table 1.

The performance as a refractometric sensor of a planar perovskite solar cell with an Indium Tin Oxide (ITO) top contact is not competitive. However, we can improve the device by replacing the ITO top electrode by a nanostructured metallic layer. Also, we move the SiO_2_ substrate (or any other substrate, including flexible materials) behind the non transparent gold electrode to expose the front surface to the analyte. In this way, we mostly preserve the structure of the solar cell and act only on the surface of a flipped, almost-finished, functional device. This arrangement allows a flexible substrate, and has been used in similar devices^54–56^. The geometry and material choice for the top contact, presented in Fig. 2a, is a metallic thin layer (in our case, a silver layer with a thickness of *t*_*m*_ =150 nm), where periodic slits are etched to trigger funneling light mechanisms at selected wavelengths^57^. The sub-wavelength period of the grating, $p=375$ nm, precludes the existence of high-order diffraction modes. Consequently, the aperture is also subwavelength, having a width ${\rm{GW}}=50$ nm, and showing a high aspect ratio with a height equal to the thickness of the metallic layer (${\rm{GH}}={t}_{m}=150AS$ nm), meaning that the slit reaches the TiO_2_ buffer layer. We have used COMSOL Multiphysics as a computational electromagnetism tool. The reliability of this approach has been proved in several analysis of nanophotonic devices, involving a variety of situations and physical mechanisms^58–61^. The calculation is made in two steps. First, a linearly polarized monochromatic wave illuminates the structure from the air. The magnetic vector is oriented along the direction of the slits, i.e., perpendicular to the plane represented in Fig. 2c. Then, the energy absorbed at the active layer is calculated using the material parameters of every layer in the structure. This is a 2D simulation because we consider an extruded geometry along the direction of the slits. The period of the subwavelength grating is repeated using periodic boundary conditions. The result is the identification of several peaks in the spectral absorption, which correspond to selective resonances of the nanostructured subwavelength grating. The second step transforms this absorbed power assuming that each photon generates a hole-electron pair that is collected by the top and bottom electrodes (assuming a collection efficiency equal to 1). This combined mechanism (selective absorption + current generation), and a monochromatic illumination allow refractometric sensing. Figure 2b shows the spectral absorption at the active layer for the proposed structure and geometry. This spectral shape is clearly different from the one belonging to a conventional perovskite solar cell (see Fig. 1b), and shows three maxima in the spectral absorption, located at ${\lambda }_{{\rm{I}}}=385$ nm, ${\lambda }_{{\rm{II}}}=715$ nm, and ${\lambda }_{{\rm{III}}}=783$ nm. It shows more peaks at different wavelengths, but these three are the most relevant in the analyzed spectral range. If we compare Figs. 1b and 2b, we see a relative low value in absorption for the modified cell. The main reason for this decreased absorption is the high reflection caused by the silver non-transparent top electrode that replaces the ITO layer. Since our goal is not energy harvesting, instead of enhancing the total photo-generated current generated by a broadband spectrum, we aim to increase the variation of the photo-current delivered by the device with respect to a change in the index of refraction. The device is illuminated with a monochromatic laser source centered at one of the maximum absorption wavelengths. This increases the variation of the current when changing the index of refraction. The next optimization step is to find the geometry of the apertures that maximizes the short-circuit current of the solar cell at each peak. When done, this optimization will provide higher values in absorption for each wavelength, as we will show in the following section (see also Fig. 3).

To understand the origin of each peak, we plot the Poynting vector and the electric field vector maps (see inset in Fig. 2a,c). The observed maxima have different field distributions along the structure, where resonances II and III generate stronger fields within the active layer. The peak at ${\lambda }_{{\rm{I}}}$ is related to plasmonic resonances caused by the periodic narrow slit. This origin is supported by the presence of hot spots in the electric field distribution at the metal/air interface (see Fig. 2c). The radiation at ${\lambda }_{{\rm{II}}}$ funnels through the gap until reaching the active layer^62^. The peak at ${\lambda }_{{\rm{III}}}$ corresponds to the strongest field distribution at the active layer. However, as we will see when analyzing its behavior with respect to the index of refraction of the analyte, this peak does not shift significantly in wavelength when changing the index of refraction, and should not be caused by a plasmonic resonance but by selective transmission through the apertures^62^.

**Table 1:** Optimized geometrical parameters of the low-order diffraction grating.

*λ* [nm]	label	*p* [nm]	GW [nm]	GH = *t*_*m*_ [nm]
385	# I	375	80	125
715	# II-narrow	375	40	140
	# II-wide	375	150	110
783	# III	375	75	140.

### Optimization

We focus on engineering an opto-electronically interrogated device able to sense changes in the index of refraction of the analyte. We will show the geometry of the design and the operation wavelengths where the proposed system provides the largest variation of the electric signal with respect to the index of refraction of the environment. In some cases, the spectral shift of the optical response of the optimized geometry allows to operate the device using spectral interrogation techniques.

The common geometry and material arrangement of a perovskite solar cell can be seen in Fig. 1a. This basic design is modified at the top surface: the ITO transparent electrode is replaced by a nanostructured metallic layer (see Fig. 2a). When including it on top of the active persovskite layer, the absorption drops. It decreases because the metallic coating is highly reflective and also dissipates energy causing losses. However, at some selected wavelengths, the absorption at the active layer, and consequently the output signal delivered by the cell, increases due to this subwavelength grating. The slits grooved on the metallic coating generate funneling and plasmonic resonances that selectively increase the amount of energy reaching the perovskite layer. The analysis of the system requires the definition of a function of merit for optimization purposes. In this case, we select the amount of energy absorbed by the perovskite active layer as the function of merit. The reason for this choice is the direct relation between the signal delivered from the cell and the absorption at the perovskite layer. The evaluation of the optical parameters is made using the high-frequency module of the multiphysic computational package Comsol Multiphysics. Due to the geometry selected in this analysis, that is an extruded 2D arrangement, the calculation can be made in 2D, allowing a faster and more reliable optimization. The structure is excited with a plane wavefront under normal incidence conditions, and having a total irradiance of 50 mW/cm^2^. The state of polarization is perpendicular to the extruded direction (the electric field vector is contained within the plane where the electric field maps in Fig. 2c are plotted, TM polarization). Periodic boundary conditions are set on both sides of the unit cell to represent an infinite array of nanoslits. Each portion of the structure is defined geometrically and optically using its complex refractive index. The photo-generated current is calculated through the absorption of the incident radiation at the active layer.

The optimization is made by a parametric sweeping of the geometry of the nanostructure (GW and GH). In practice, we maximize the optical absorption at the solar cell active layer, ${A}_{{\rm{p}}{\rm{e}}{\rm{r}}{\rm{o}}{\rm{v}}}$. We maximize it for those wavelengths generating a large absorption at the active layer because they will generate a large electric signal, which is better suited to sense environmental changes. After optimizing this variable, we have obtained the spectral absorption for the three wavelengths of interest. The actual parameters that maximize absorption are given in Table 1 for each wavelength. So, we have maximized the amount of energy absorbed at the perovskite active layer through the combination of plasmonic resonances, funneling effects and selective transmission, by varying the geometrical parameters of the slits. In Figure 3 we represent the maps of the absorption at the perovskite active layer, ${A}_{{\rm{perov}}}$, in terms of the width (GW) and height (GH) of the slits, for each wavelength of the resonances identified previously (see labels in Table 1).

## Results and Discussion

**Figure 4:**
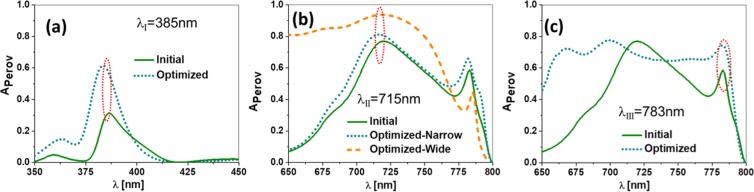
Spectral absorption in the active layer for the optimum geometrical parameters at the three resonant wavelengths. the absorption in the active layer for the design with the arbitrary selected geometry is included for each case to show the effect of the optimization process.

We can see that the maximum absorption, ${A}_{{\rm{perov}}}=\mathrm{94 \% }$ (see Fig. 3b), is obtained for a wide slit at ${\lambda }_{{\rm{II}}}=715$ nm. The colormap of these plots has been normalized independently for each wavelength. Obviously, to compare the results among them, it is necessary to consider the maximum value for each one. Figure 4 shows the spectral absorption of the optimized devices for each wavelength previously selected (see Fig. 3 and Table 1). We see how the absorbed power is significantly larger after optmization.

**Figure 5:**
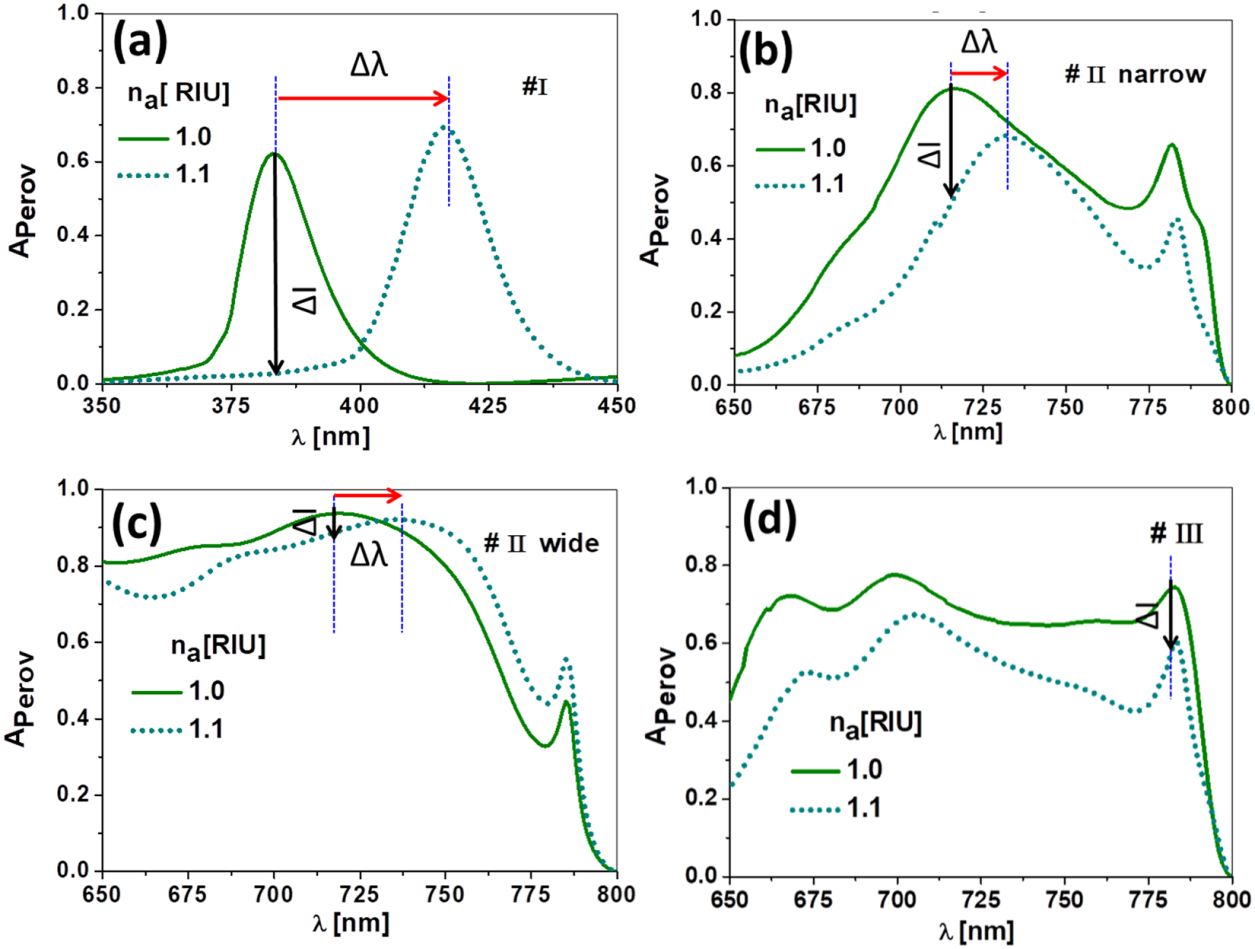
Spectral absorption for the four optimized designs at the three resonant wavelengths. These absorptions are calculated for two values of the index of refraction of the analyte, ${n}_{a}\mathrm{=1.0}$ and 1.1. The vertical blue dotted line represents the wavelength of resonance for ${n}_{a}\mathrm{=1.0}$, and 1.1. Δ*λ*, is the spectral shift of the resonance when varying the index of refraction, and Δ*I* shows the variation in absorption at the perovskite active layer.

To explore the capabilities of the proposed device for spectral and opto-electronic interrogation methods, we have evaluated the spectral absorption for each optimized case, and for two different values of the index of refraction, *n*_*a*_ = 1.0 and 1.1. This range is much larger than the expected variations in the index of refraction, and explores how this device behaves when adding nebulized liquids in the atmosphere, or when the analyte is under extreme values of pressure and temperature^38^. Besides, this extended range reveals better how the spectral absorption changes with respect to the index of refraction (as shown in Fig. 5). In our calculation, we have considered the gases as non-absorptive (meaning a negligible value of the imaginary part of the index of refraction). We have also neglected the contribution of scattering, but this simplification should be raised if nano- and micrometer size particles were taken into account^63^. In Fig. 5, we show how each design undergoes a spectral shift, Δ*λ*, and absorption intensity change, Δ*I*, as the refractive index changes. In this last case, a variation in the absorption at the active layer translates in a change in the photo-generated current, and hence in responsivity. A larger Δ*λ*, or Δ*I*, means a higher sensitivity because it generates a larger measurable change when the index of refraction varies. However, the measurement method is different: Δ*λ* is obtained through a spectral interrogation scheme, and Δ*I* is measured using an opto-electronic set-up where the electric signal delivered by the cell is processed by an external circuit. In this case, the electronic output signal of the device senses the variation of the refractive index, which means an opto-electronic interrogation.

We simulate our device’s performance to measure the index of refraction of gases (with index of refraction very close to 1.0). The system works with a monochromatic light source tuned to the selected resonant wavelength of each one of the designs (${\lambda }_{{\rm{I}}},{\lambda }_{{\rm{II}}-{\rm{narrow}}}={\lambda }_{{\rm{II}}-{\rm{wide}}},$ or ${\lambda }_{{\rm{III}}}$). To properly compare the four designs, the light source illuminates the device with the same irradiance of 50 mW/cm^2^. This value is achievable using regular diode lasers. At the same time, the spectral width of the resonance is large enough compared with the typical linewidth of commercial laser diodes (well below 0.1 nm), to consider the light source as purely monochromatic (as a delta function in *λ* that samples the absorbed spectral). This irradiance generates a photo-current, ${I}_{{\rm{sc}}}$, that is retrieved from the cell and delivered to an electronic read-out circuit. When changing the index of refraction of the analyte, the current varies, and so does the delivered signal. The photo-current is used to calculate the responsivity, R, which is defined as^64^:

1 $${\rm{R}}(\lambda )=\frac{{I}_{{\rm{sc}}}}{{P}_{{\rm{input}}}(\lambda )},$$

where ${I}_{{\rm{sc}}}$ is the ideal photo-generated short-circuit current, and ${P}_{{\rm{input}}}(\lambda )$ is the incident light power that is delivered by the monochromatic source.

**Figure 6:**
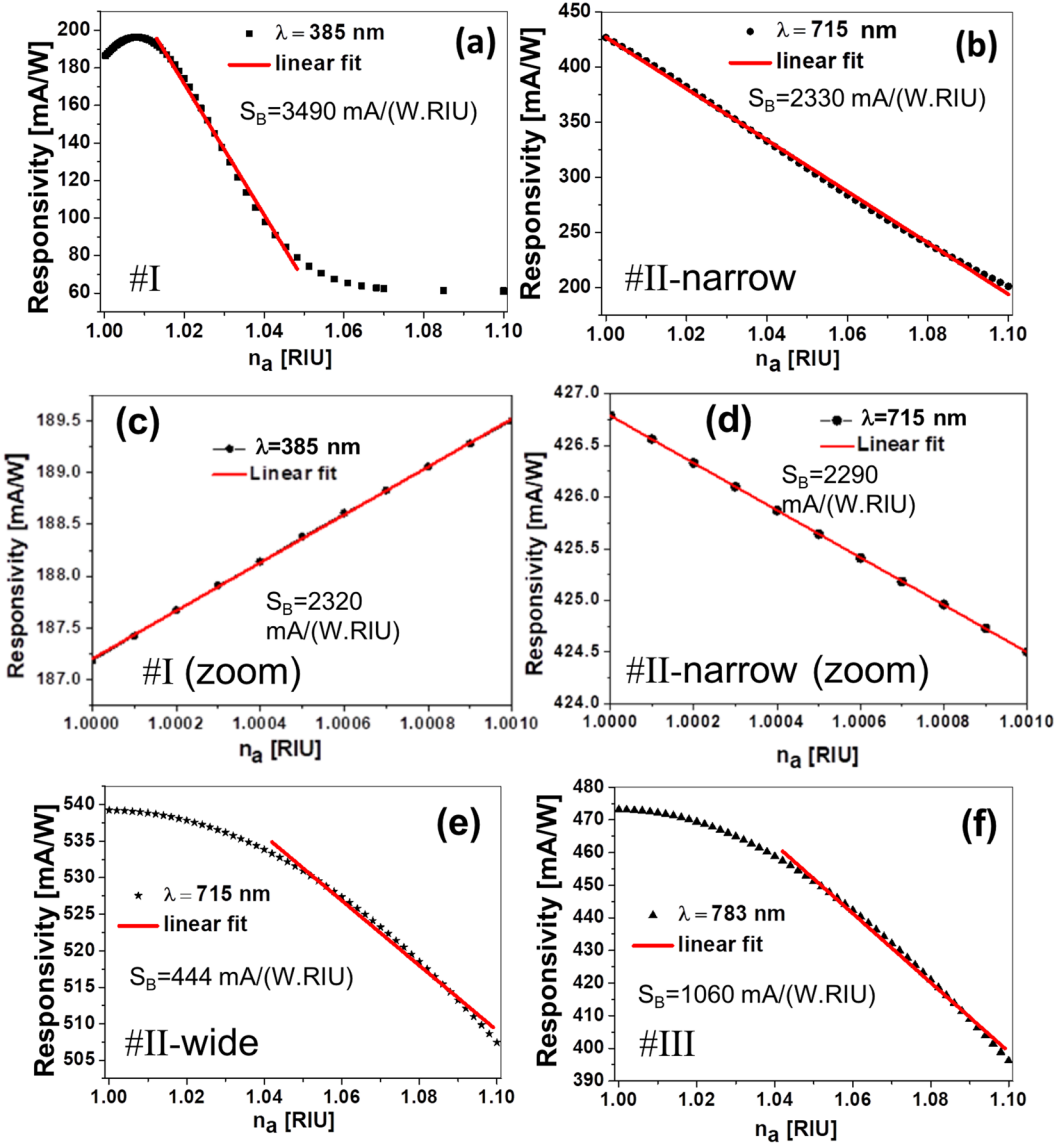
Variation of the responsivity, *R*, as a function of the index of refraction of the analyte, *n*_*a*_, for the four optimum design selected in this paper. Plots (**c**,**d**) zoom in the region ${n}_{a}\in [1.000,1.001]$, where the index of refraction of gases typically vary, for designs # I and # II-narrow. The red lines limit the range in the index of refraction where the variation in the current can be linearly fitted, meaning a constant value of the sensitivity of the device, ${S}_{B}$.

The change in the responsivity with respect to *n*_*a*_ is presented in Fig. 6. As pointed out in the introduction section, the sensitivity of this sensor can be defined as the variation of R with respect to the index of refraction of the analyte, *n*_*a*_^49,50,65^:

2 $${S}_{B}=\frac{\partial {\rm{R}}}{\partial {n}_{a}},$$

and the Figure of Merit (FOM) is also redefined in terms of the responsivity, *R*^49,50^:

3 $${\rm{FOM}}=\frac{{S}_{B}}{R}\mathrm{}.$$

These definitions are adaptations of those applicable to plasmonic sensors interrogated in wavelength or angle of incidence, as it happens with Kretschmann and Otto configurations.

The evaluation of the sensitivity for the four designs presented in this paper, can be made using Eq. (2). Table 2 summarizes the performance of the four designs in terms of sensitivity and FOM (Eqs. (2) and (3)). This table also includes the variations $\varDelta \lambda$ and $\varDelta {A}_{{\rm{perovs}}}$, that are applicable for a spectral interrogation method, or an opto-electronic technique, respectively. Our proposal is to measure using electric parameters, where ${I}_{{\rm{s}}{\rm{c}}}$ is proportional to ${A}_{{\rm{perov}}}$, and therefore, the sensitivity is given using its definition in terms of ${\rm{R}}$. Figure 6 plots the responsivity for the four designs treated in this contribution. For the first peak at ${\lambda }_{{\rm{I}}}$, the change in photo-current when varying the index of refraction is large, in accordance with a 60% change in the absorption at the active layer (see Fig. 5a and Table 2). This means a high sensitivity to refractive index changes, but results in a limited dynamic range since the current decays sharply (see Fig. 6a). The spectral absorption of the second peak at ${\lambda }_{{\rm{I}}{\rm{I}}}$ (see 6a, # II-narrow case), generates a smaller spectral shift while maintaining a significant variation in intensity. Its responsivity is high and linear within the studied range in the index of refraction (see 6b). This expanded range in linearity is desiderable, and compared to the previous design at ${\lambda }_{{\rm{I}}}$, makes this design operationally more robust and stable. Figure 6c,d contains a detailed zoom of the responsivity in the range ${n}_{a}\in \mathrm{[1.000,1.001]}$ that comprises the expected variations in the index of refraction of air or controlled atmospheres. We have selected designs # I and # II-narrow because they show a better behavior (higher ${S}_{B}$). We can see, that these designs, even in this narrow range, provide competitive values of sensitivity. The corresponding performance values are also shown in Table 2. The spectral absorption for designs # II-wide, and # III presents a lower value of $\Delta I$ (see Fig. 5c,d) that corresponds with a lower value of sensitivity (see Fig. 6c,d, and Table 2). Even more, in the design # III at ${\lambda }_{{\rm{III}}}\mathrm{=783}$ nm, the spectral shift, $\Delta \lambda$, is negligible and precludes its operation using spectral interrogation methods. This fact is in agreement with the physical interpretation of this peak as a selective transmitance through the slits^62^. The FOM results summarized in Table 2 are moderate compared with previously reported figures^1–4,6–9^.

**Table 2:** Performance parameters for the four designs.

Design type	Δλ_r*es*_[nm]	Δ*A*_perov_[%]	*S*_*B*_[mA(W.RIU) ^−1^]	FOM[RIU ^−1^]	FOM ^*^[RIU ^−1^]
Planar cell	NA	1.4	70	0.15	NA
# I	33.2	60.0	3490	17.7	17700
# I (zoom)	—	—	2320	12.2	12200
# II-narrow	17.5	31.8	2330	5.5	5450
# II-narrow (zoom)	—	—	2290	5.4	5380
# II-wide	16.5	5.4	444	0.82	824
# III	0	15.0	1060	2.24	2240

FOM for angular and spectral interrogation systems is defined as the ratio between sensitivity, ${S}_{B}$, and the capability of the system to distinguish a variation in the signal. This resolution capability is given as the full width at half maximum (FWHM) of the measured lineshape (in terms of angle or wavelength). The case treated in this paper generates an electric signal, the current delivered by the perovskite cell, that senses the variation in the index of refraction. This current will be affected by noise and fluctuations due to the intrinsic variations of the sensor, and related with the stability and noise level of the light source, the detector, and the signal acquisition electronics. Most of these variations can be accounted for and controlled. In the end, all this uncertainties are summarized in a variation of the responsivity, $\Delta R$. which plays the same role as the FWHM used to define FOM for spectral or angular interrogation. A modification of the figure of merit adapted to this measurement procedure would be defined as:

4 $${{\rm{FOM}}}^{\ast }=\frac{{S}_{B}}{\Delta {\rm{R}}},$$

where the ^*^ superscript is given here to distinguish this definition from the one given in Eq. (3). In the practical case that we treat here, when comparing the denominator in Eq. (3) with $\Delta {\rm{R}}$, we can conclude that the definition given in Eq. (3) clearly underestimates the FOM for our system.

From the definition of responsivity (Eq. (1)), we can derive its uncertainty, ${\sigma }_{{\rm{R}}}$, that can be used in Eq. (4), considering $\Delta {\rm{R}}={\sigma }_{{\rm{R}}}$, as:

5 $$\Delta R={\sigma }_{{\rm{R}}}=\sqrt{{\left(\frac{\partial {\rm{R}}}{\partial {I}_{{\rm{sc}}}}\right)}^{2}{\sigma }_{{I}_{{\rm{sc}}}}^{2}+{\left(\frac{\partial {\rm{R}}}{\partial {P}_{{\rm{input}}}}\right)}^{2}{\sigma }_{{P}_{{\rm{input}}}}^{2}}=\sqrt{\frac{{I}_{n}^{2}}{{P}_{{\rm{input}}}^{2}}+{{\rm{R}}}^{2}{\left(\frac{\Delta {P}_{{\rm{input}}}}{{P}_{{\rm{input}}}}\right)}^{2}},$$

where $\varDelta {P}_{{\rm{input}}}/{P}_{{\rm{input}}}$ describes the stability in power of the illuminating light source. Assuming and accurate measurement of the current, we have taken its uncertainty as the noise in intensity of the photodetector ${\sigma }_{{I}_{{\rm{sc}}}}={I}_{{\rm{n}}}$. The uncertainty of the illuminating power has been written in terms of the relative power stability of the source, ${\sigma }_{{P}_{{\rm{input}}}}=\varDelta {P}_{{\rm{input}}}$. The device described in this contribution is quite close to a photodetector. Therefore, as it has been already proposed for perovskite based detectors^53^, we should consider photodetection quality parameters to assess the performance of the device. Among these parameters, the noise equivalent power, NEP, is defined as ${\rm{NEP}}={I}_{n}/{\rm{R}}$, and evaluated for perovskite detectors as ${\rm{NEP}}\mathrm{=4.6}\times {10}^{-12}$ W at a wavelength $\lambda =700$ nm^53^. When taking the worst-case scenario with the largest value in responsivity evaluated in this paper ($R=539$ mA/W at $\lambda =715$ nm), we obtain an estimation of the maximum noise in intensity: ${I}_{n}={\rm{NEP}}\times R=2.5\times {10}^{-3}$ pA. Taking into account this very small value of ${I}_{n}$, we find that the largest contribution to the variation in responsivity is coming from the second term in Eq. (5). This means that, after neglecting the first term within the square root in Eq. (5), we obtain

6 $$\Delta {\rm{R}}={\sigma }_{{\rm{R}}}\simeq ={\rm{R}}\frac{\Delta {P}_{{\rm{input}}}}{{P}_{{\rm{input}}}},$$

meaning that the relative variation in responsivity is equal to the relative variation in input power: $\Delta {\rm{R}}/{\rm{R}}=$ ${\Delta {\rm{P}}}_{{\rm{input}}}{/{\rm{P}}}_{{\rm{input}}}$. Using this approximation and combining Eqs. (3) and (4), it is possible to obtain a relation between ${\rm{FOM}}$ and ${{\rm{FOM}}}^{\ast }$ as:

7 $${{\rm{FOM}}}^{\ast }={\left(\frac{\Delta {P}_{{\rm{input}}}}{{P}_{{\rm{input}}}}\right)}^{-1}\times {\rm{FOM}},$$

showing that both performance parameters are proportional, and the proportionality constant depends on the stability in power of the light source, i. e., a more stable light source means a larger multiplicative term in Eq. (7). A value of $\Delta {P}_{{\rm{input}}}/{P}_{{\rm{input}}}{\mathrm{=10}}^{-3}$ is attainable with the current laser diode technology. This means that ${{\rm{FOM}}}^{\ast }\mathrm{=1000}\times {\rm{FOM}}$. This previous discussion makes possible a conservative evaluation of $\Delta {\rm{R}}$ that we have included in the last column of Table 2, where we have found that ${{\rm{FOM}}}^{\ast }$ reaches a significant maximum value of ${{\rm{FOM}}}^{\ast }\mathrm{=17700}\,{{\rm{RIU}}}^{-1}$, which is competitive with existing technologies. Within the range ${n}_{a}\mathrm{=[1.000,1.001]}$, this value is FOM^*^ = 12200. This value would mean a capability of measuring change in the index of refraction as low as $8\times {10}^{-5}$ RIU, that may account for variations in the index of refraction of air^5,14,34–36,39,45^.

## Conclusions

The device proposed in this paper is able to sense variations of the index of refraction of the analyte using the electric signal delivered by a modified perovskite solar cell. Although a standard perovskite cell could work as an unsophisticated refractometric sensor, its design still allows improvement for this application. To take advantage of perovskite’s cell technologies, instead of designing a complete pervoskite sensor, we have just modified the front surface of the cell. This modification consists of a regular pattern of nanoslits engraved on a silver layer. This nanostructure substitutes the front ITO electrode in a flipped configuration where the substrate moves towards the back contact, exposing the analyte to the front metallic layer. This modified arrangement is responsible for the spectral selectivity of the system. Besides, the geometrical parameters are selected within feasible and fabricable ranges to maximize the signal obtained from the device. This optimization considers the analyte as a gas medium with index of refraction close to 1. The device is illuminated with a monochromatic source tuned at one of the resonant wavelengths. The short-circuit current generated by the device feeds the measurement electronics to provide a signal that senses the changes in the index of refraction of the analyte. We have adapted the definitions of sensitivity and FOM to the case of an opto-electronic interrogation method. These results help us to identify which of the resonant wavelengths and designs works better as a refractometric sensor. The evaluation of the FOM shows a relevant value as large as 17.7 RIU ^−1^ that, however, it is still moderate when compared with existing technologies. Moreover, this system gets rid of complicated, voluminous, and expensive elements used when spectral interrogation is necessary. On the other hand, if the definition of the figure of merit uses photodection parameters, the values of FOM should be replaced by a modified FOM ^*^ where the resolution in responsivity is given in terms of the limitations of the sensor taken as a photodetector. When doing this, we find a value of this modified figure of merit as large as ${{\rm{FOM}}}^{\ast }$ = 17700 ${{\rm{RIU}}}^{-1}$ (${{\rm{FOM}}}^{\ast }$ = 12200 ${{\rm{RIU}}}^{-1}$ within the range ${n}_{{\rm{a}}}=\mathrm{[1.000,1.001]}$). From the previously obtained results, it seems that designs # I and # II-narrow are the most promising alternatives. In any case, the choice of one of the four designs will depend on the availability of light sources, the reliability of the fabrication, and the selection of a higher performance in terms of the FOM (or FOM^*^), or an expanded linear behavior.

Summarizing the finding of this paper, we are proposing a refractometric sensor that modifies a perovskite solar cell. This modification takes the form of a subwavelength metallic grating acting as the front contact of the cell. All the system (light source, sensor, and acquisition electronics) can be packed together within a tiny volume allowing miniaturization. The detection uses the electric signal delivered by the cell. This approach does not require any goniometer or spectrometer, making the device easier to operate. When considering a modified figure of merit, ${{\rm{FOM}}}^{\ast }$, to describe the behavior of the device, the reported values are very competitive with existing technologies.
